# Supplementary material for: Towards unified AI-driven fracture mechanics: the extended deep energy method (XDEM)
Source: Nat Commun. 2026 Aug 15;17:8492. doi: 10.1038/s41467-026-76748-1 (PMC13484471; doi:10.1038/s41467-026-76748-1)
Supplement: Supplementary file 3 [file 41467_2026_76748_MOESM3_ESM.zip › pic/Results/Crack_propagation/Initiation/crack_initiation.pdf]

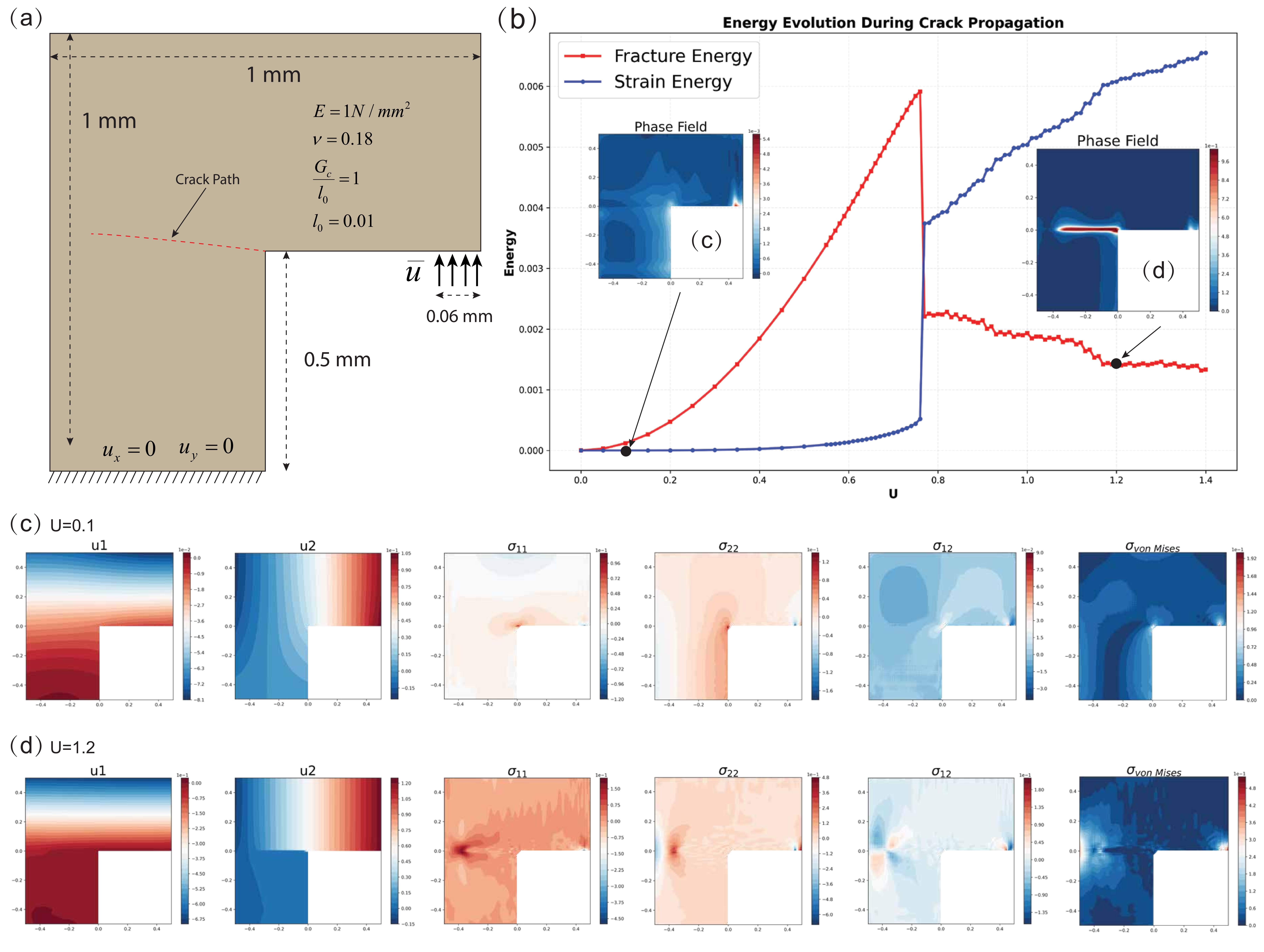
(a)
1 mm
1 mm
$E = 1N/mm^2$
$\nu = 0.18$
$\frac{G_c}{l_0} = 1$
$l_0 = 0.01$
Crack Path
$\bar{u}$
0.06 mm
0.5 mm
$u_x = 0$ $u_y = 0$
(b)
Energy Evolution During Crack Propagation
Fracture Energy
Strain Energy
Phase Field
(c)
Phase Field
(d)
Energy
U
(c) U=0.1
u1
u2
σ_{11}
σ_{22}
σ_{12}
$\sigma_{von\ Mises}$
(d) U=1.2
u1
u2
σ_{11}
σ_{22}
σ_{12}
$\sigma_{von\ Mises}$
